# Glaucoma in a Siberian Tiger (*Panthera tigris altaica*) Maintained Under Human Care: A Case Report

**DOI:** 10.3390/ani16111647

**Published:** 2026-05-28

**Authors:** Gábor Lorászkó, András Dobos, András Dobos, Pál Szekér, Péter Tóth-Almási, Péter Sótonyi, László Ózsvári

**Affiliations:** 1 Department of Anatomy and Histology, University of Veterinary Medicine Budapest, 1078 Budapest, Hungary; loraszko.gabor@univet.hu (G.L.); sotonyi.peter@univet.hu (P.S.); 2 Kiséri Veterinary and Laser Eye Clinic, 6600 Szentes, Hungary; szentesiallatorvos@gmail.com (A.D.S.); d990new@gmail.com (A.D.J.); 3 Kittenberger Kálmán Zoo & Botanical Garden, 8200 Veszprém, Hungary; dr.szeker@gmail.com (P.S.); tothpetya@yahoo.com (P.T.-A.); 4 Department of Veterinary Forensics and Economics, University of Veterinary Medicine Budapest, 1078 Budapest, Hungary

**Keywords:** felids under human care, glaucoma, intraocular pressure, tonometry, fundus imaging, ultrasonography, focal seizures, veterinary ophthalmology, zoo animal medicine

## Abstract

Primary open-angle glaucoma (POAG) is rarely recognized in large felids, yet it may cause severe vision loss and chronic pain. This case report presents the ophthalmological evaluation of a four-year-old Siberian tiger (*Panthera tigris altaica*) maintained under human care that exhibited signs of visual impairment and altered sensorium. Under neuroleptic analgesia, clinical findings included bilateral convergent strabismus, persistent mydriasis, pathologically elevated intraocular pressure (48–52 mmHg), extensive map-like retinopathy, and focal retinal degeneration, supporting a diagnosis of advanced-stage bilateral POAG with irreversible vision loss. Pupillary light reflexes were absent, while fluctuations in pupil size were attributed to ketamine. Although medical and surgical options used in domestic cats may be applicable to non-domestic felids, frequent topical treatment was impracticable and unsafe, vision-preserving surgery was no longer indicated, and advanced diagnostics and specialist ophthalmic surgery were unavailable on site. The therapeutic goal was therefore shifted from vision preservation to long-term pain surveillance and welfare maintenance, including housing adaption and a defined threshold for humane euthanasia. Two types of neurological episodes were also documented as secondary findings; however, their causal relationship with the ocular disease could not be established without advanced neuroimaging. This case highlights the need for accessible on-site or referral-based ophthalmic diagnostics and surgical capacity in zoo settings.

## Simple Summary

This case report presents the ophthalmological examination of a four-year-old female Siberian tiger (*Panthera tigris altaica*) that had arrived at the zoo a few months earlier and had displayed atypical behavior from the outset. The sudden, violent behavior, fear, aggression, and disorientation of the animal gave the impression that she was probably blind. Examinations performed under general anesthesia confirmed markedly elevated intraocular pressure in both eyes, together with fundic lesions indicative of advanced glaucoma. Pupillary light reflexes were absent; the fluctuations observed in pupil size were attributable to the pharmacological effects of ketamine. Because of the limitations of the examination site, surgical intervention was not performed. In addition to the ocular findings, two different types of neurological episodes were documented: generalized tonic–clonic seizures during recovery from anesthesia, and brief stereotyped episodes—consistent with focal seizures rather than true behavioral rage—that had been observed repeatedly since the animal’s arrival at the zoo. A direct causal link between either event type and the ocular disease could not be established without advanced neuroimaging. Taking animal-welfare considerations into account, the tiger was returned to her original environment, where she was able to move around safely despite her visual loss.

## 1. Introduction

Glaucoma is a neurodegenerative process involving the progressive degeneration of the optic nerve head and retinal ganglion cells (RGC), often—though not invariably—accompanied by an elevated intraocular pressure (IOP) [1]. The disease ultimately leads to irreversible blindness. In the domestic cat, the physiological IOP is approximately 15–25 mmHg [2]. The modern understanding of aqueous-humor dynamics in felids, including the identification of the alternative uveoscleral outflow pathway and its species-specific characterization, derives from the foundational physiological work of Bárány [3]. Primary open-angle glaucoma (POAG) is a well-documented condition in humans and dogs, frequently underpinned by genetic mutations that cause increased resistance to aqueous-humor outflow despite a macroscopically open iridocorneal angle [4,5].

The literature on glaucoma in non-domestic felids is limited. Accordingly, the pathophysiology, genetic background, and optimal therapeutic protocols for glaucoma in large felids remain insufficiently explored areas in veterinary ophthalmology. The ophthalmological examination of large felids poses a significant diagnostic challenge due to the requirement for chemical immobilization and the specialized equipment needed for clinical evaluation [6]. Drugs used during immobilization, particularly the dissociative anesthetic ketamine, can significantly influence intraocular pressure and pupil size, complicating the interpretation of clinical findings [7].

The objective of this case report is to provide a detailed presentation of the clinical course of POAG diagnosed in a Siberian tiger (*Panthera tigris altaica*), the diagnostic difficulties encountered, and the potential associations with neurological signs.

## 2. Case Presentation

### 2.1. Case History and Clinical Examination

On 27 October 2024, a 4-year-old female Siberian tiger (*Panthera tigris altaica*), born in France and transported to Hungary four months earlier, was examined at the Kittenberger Kálmán Zoo and Botanical Garden in Veszprém. Maintained under human care, the animal had exhibited atypical behavior, brief stereotyped episodes initially interpreted by the keepers as aggressive outbursts, disorientation, and suspected visual impairment since her arrival. During the four-month acclimatization period following the animal’s arrival at the zoo and preceding the ophthalmological examination, the tiger did not receive any analgesic, sedative, tranquilizer, or other systemic medication. The observed behavioral changes were initially attributed to environmental stress and adaptation to the new enclosure. This pharmacologically neutral baseline was relevant for the subsequent ophthalmological examination, because α_2_-adrenergic agonists have been reported to lower intraocular pressure in domestic cats [8], a finding that could, if present, mask the magnitude of pre-existing ocular hypertension. The absence of prior pharmacological exposure therefore supports the diagnostic validity of the markedly elevated intraocular pressure subsequently recorded in both eyes. The clinical presentation was characterized by a diminished response to environmental stimuli and a lack of visual orientation, indicating advanced bilateral ocular or neurological involvement. Consequently, the institution’s management requested a consultation with veterinary ophthalmologists to establish a diagnosis. The examination lasted two and a half hours and began with remote observation in a quiet environment, followed by a detailed ophthalmological evaluation under general anesthesia.

The clinician’s approach with the pneumatic remote drug delivery system generated an acoustic stimulus that elicited an auditory orientation reflex, namely head-turning, but no visual avoidance response (Video S1). The tiger remained stationary on the elevated platform until the mechanical impact of the dart. An immediate escape reaction followed the impact, after which the animal returned to the 120 cm high elevated resting platform (Video S2).

**Figure 1:**
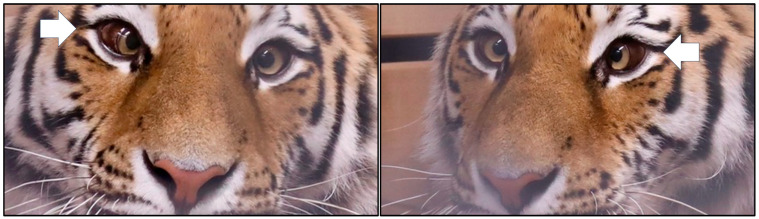
Bilateral convergent strabismus (esotropia) in the examined Siberian tiger (*Panthera tigris altaica*). Note: Arrows indicate the affected eyes.

During remote observation under adequate lighting, the tiger did not follow the movement of personnel with her gaze and showed no reaction to visual stimuli, including camera flashes and lights being turned on and off, relying entirely on olfactory and auditory cues. In response to auditory stimuli, rapid cycles of involuntary muscle contraction and relaxation, consistent with muscle fasciculations, were observed in the cervical musculature. Remote observation revealed pronounced bilateral convergent strabismus, or esotropia (Figure 1). In felids, this clinical sign may reflect disruption of the visual pathways, involvement of cranial nerves III, IV, or VI, or intracranial processes affecting the ocular motor system [9].

Objective weighing of the subject was not feasible either before or during the anesthetic procedure. Before anesthesia, the tiger’s visual impairment precluded reliable guiding of the animal onto a walk-on scale, because the blind subject did not respond consistently to directional cues from keepers. During anesthesia, safety considerations required that the tiger remain on her elevated resting platform inside the holding cage or night house; no scale was available in this area. The body weight was therefore visually estimated at approximately 160 kg by two of the attending zoo veterinarians (P. Szekér and P. Tóth-Almási), and all drug doses were calculated on this basis [6,9]. At onset (T = 0 min), 5 mg medetomidine (0.031 mg/kg) and 250 mg ketamine (1.562 mg/kg) were administered intramuscularly (IM). At T = 23 min, 1.5 mg medetomidine (0.009 mg/kg), 30 mg butorphanol (0.188 mg/kg) and 200 mg ketamine (1.250 mg/kg) were administered IM. At T = 32 min, a further 1.5 mg medetomidine (0.009 mg/kg) and 200 mg ketamine (1.250 mg/kg) were given IM. Reversal was induced at T = 59 min with 50 mg atipamezole (0.312 mg/kg) IM. Initial signs of recovery, including ear movements and head lifting, were observed at approximately T = 69 min.

Throughout the anesthesia, the tiger was maintained in lateral recumbency on her elevated resting platform inside the holding cage. Supplemental oxygen was not available under these field conditions. Intravenous access was established in the left lateral saphenous vein using an 18G Vasofix^®^ Braunüle^®^ peripheral IV catheter (B. Braun Melsungen AG, Melsungen, Germany), and Ringer’s lactate solution was delivered by a veterinary infusion pump (Hawkmed HK-100VET; Shenzhen Hawk Medical Instrument Co., Ltd., Shenzhen, China) at 800 mL/h (5 mL/kg/h). Physiological monitoring during anesthesia comprised continuous auscultation of heart rate with a stethoscope; continuous monitoring of peripheral oxygen saturation (SpO_2_) and pulse rate with a portable pulse oximeter (Masimo Rad-5; Masimo Corporation, Irvine, CA, USA) fitted with a lingual sensor; visual observation of respiratory rate through thoracic excursions; and intermittent measurement of rectal temperature. Capnography, electrocardiography and inhalational anesthesia were not available on site; non-invasive manual monitoring was therefore employed as the standard of care for field-based chemical immobilization of large non-domestic felids [6,10,11,12].

During the recovery phase, before and during the transition to full consciousness, the animal experienced several generalized tonic–clonic seizures, each lasting approximately one minute. These episodes were characterized by lateral recumbency, opisthotonus, and paddling, or swimming movements, consistent with grand mal epilepsy, accompanied by involuntary urination and defecation (Video S3). These were the only generalized tonic–clonic seizures ever observed in this animal; no such events had been reported by the keepers before the anesthesia, nor were any observed in the subsequent days.

In addition to the generalized tonic–clonic seizures observed only during recovery, the keepers and veterinarians at the holding zoo reported recurrent brief episodes of an entirely different character that had been observed repeatedly since the animal’s arrival at the zoo four months before the anesthesia. A representative episode was recorded on the day after the examination (Video S4). These episodes lasted approximately 15 s and were characterized by tremor, head-striking against the cage bars, and motor incoordination, but without falling, clawing, or pawing or striking movements typical of a true behavioral rage response. Because the animal was permanently housed indoors owing to her blindness, direct observation by the keepers was limited to feeding and cleaning times; the precise frequency of these episodes was therefore not known, but they were reported by the keepers as occurring on multiple occasions and without an identifiable external trigger. Their brief, stereotyped character, the preservation of postural tone, and the presence of head-directed motor automatisms are more consistent with focal (partial) seizures, possibly of limbic origin, than with a behavioral or aggressive episode [13].

### 2.2. Ophthalmological Examination Findings

*Physical examination findings:* On digital palpation performed during the examination under anesthesia, both eyeballs felt distinctly firm. The eyelids were intact, the sclera was undamaged, and the third eyelid was normal. The cornea was free of vascularization and ulceration, the anterior chamber was clear, the iris was well-pigmented with no visible lesions, and the pupillary margin was mobile. Both pupils were circular and so persistently dilated under anesthesia that no topical mydriatic agent (e.g., tropicamide or atropine) was required for the subsequent ophthalmological examination. This was considered atypical for tigers, in which pharmacological mydriasis is usually required for complete anterior-segment and fundus assessment. In *Panthera* species, including the tiger, the pupil is circular rather than vertically slit-shaped, as is characteristic of ambush predators among smaller felids [14]. To the authors’ knowledge, subspecies-specific reference values for photopic and maximally dilated pupillary diameter in the Siberian tiger are not available in the peer-reviewed literature. For anatomical context, the only published ocular biometric study in adult tigers (*Panthera tigris*) reports a mean axial globe length of 29.36 ± 0.82 mm, an anterior chamber depth of 7.00 ± 0.74 mm, and a crystalline lens thickness of 8.72 ± 0.56 mm [15].

Direct and consensual pupillary light reflexes (PLRs) were completely absent, with no response to light stimuli. Both pupils remained markedly and persistently dilated throughout the examination, with intermittent fluctuations in pupillary diameter. Clinically, both eyes showed a marked convergent position, consistent with the severe bilateral convergent strabismus described previously.

**Figure 2:**
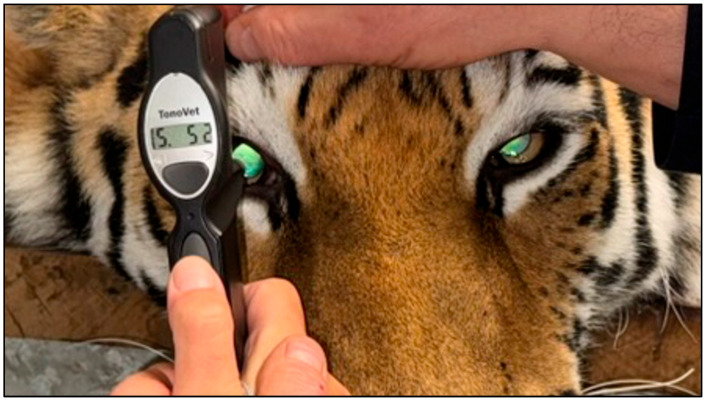
Intraocular pressure of 52 mmHg measured with a TonoVet rebound tonometer in the right eye.

*Instrumental examination findings:* Intraocular pressure was measured with a rebound tonometer (TonoVet; Icare Finland Oy, Vantaa, Finland). Repeated measurements over 30 min yielded values of 48 mmHg in the left eye and 52 mmHg in the right eye (Figure 2).

The anterior segment was evaluated by biomicroscopy using a portable Keeler PSL Classic slit lamp (Keeler Ltd., Windsor, UK). During this process, the iridocorneal angle and the pectinate ligaments were also assessed by direct observation with focal illumination and magnification, and with an indirect ophthalmoscope with a 20D condensing lens held at an extreme angle. These observations suggested an open and intact drainage angle. However, because formal gonioscopy could not be performed under the field conditions, subtle pectinate-ligament abnormalities could not be excluded. Because of the field conditions and the use of portable diagnostic equipment, integrated digital imaging was not available for these specific segments; therefore, findings were documented through detailed clinical observation.

**Figure 3:**
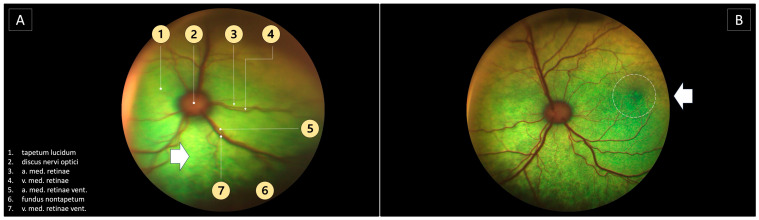
Fundus photographs of the examined Siberian tiger: right eye (**A**) and left eye (**B**). Note: In panel (**A**), the white arrow indicates a representative area of the map-like retinopathy. In panel (**B**), the white dotted circle outlines a focal degenerative retinal lesion, which is also indicated by the white arrow.

During ophthalmoscopy, mild lens opacities were noted; the lens suspensory fibers appeared intact, and the lens was positioned normally. The fundus was clearly visualized through the lens with a fundus camera (ClearView Optical Imaging System; Optibrand Ltd., LLC, Fort Collins, CO, USA). In both eyes, the optic nerve head was ophthalmoscopically unremarkable, with disk margins, color, and vascular contours within the expected range for the species. No overt cupping or excavation, peripapillary pigmented or hyper-reflective halo, or increased prominence of the lamina cribrosa pores was observed. Bilateral extensive retinopathy with a map-like pattern was observed accompanied by a focal degenerative lesion in the left eye (Figure 3).

**Figure 4:**
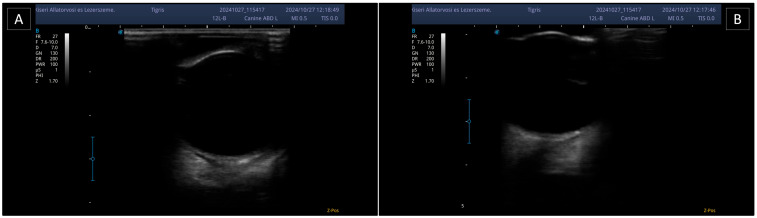
B-mode ocular ultrasonography of the right (**A**) and left (**B**) eyes of the examined Siberian tiger.

B-mode ocular ultrasonography was performed with a digital color Doppler ultrasound system (Model E2V; SonoScape Medical Corp., Shenzhen, China), using a 12L-B linear-array transducer operating at a frequency range of 3.0–17.0 MHz. The examination was specifically targeted at potential secondary causes of elevated intraocular pressure and demonstrated a normal contour of the posterior pole, with no evidence of retinal detachment, intraocular hemorrhage, periocular soft-tissue inflammation, intraocular space-occupying masses, or other abnormalities indicative of an alternative pathological process (Figure 4). Together with the clinical and tonometric findings, these results helped exclude the principal secondary causes of elevated intraocular pressure and supported the diagnosis of POAG [6,16]. The oral cavity was intact.

## 3. Discussion

Scientific reports of glaucoma in non-domestic large felids are scarce. In tigers (*Panthera tigris*), two cases have been documented: unilateral secondary glaucoma with anterior staphyloma in a 22-year-old circus-rescued male tiger with bilateral intraocular pressures of 90 and 88 mmHg [17], and unilateral congenital glaucoma with goniodysgenesis in an 8-month-old female tiger with a rebound-tonometry value of 70 mmHg in the affected eye [18]. In lions (*Panthera leo*), secondary glaucoma has been reported in association with anterior uveal neoplasia, including a buphthalmic globe caused by anterior uveal melanoma in a 19-year-old lioness [19], and diffuse iris melanoma with buphthalmos, corneal edema, and an intraocular mass occupying the ciliary cleft in a 12-year-old male African lion [20]. Scientific case reports of glaucoma in the snow leopard (*Panthera uncia*), leopard (*Panthera pardus*), and puma (*Puma concolor*) could not be identified in the indexed veterinary literature available to the authors.

Normative intraocular-pressure data for *Panthera tigris* are limited to the study of Owens et al. [14], in which 33 eyes of 17 clinically normal adult tigers, with subspecies not specified, under general anesthesia yielded a mean ± SD applanation-tonometry IOP of 14.7 ± 2.69 mmHg. Subspecies-specific ophthalmic reference data for the Siberian tiger (*Panthera tigris altaica*) are not available in the scientific literature to the authors’ knowledge; therefore, comparison between the present case and the published adult-tiger data must be interpreted as a cross-subspecies comparison.

To contextualize the rebound-tonometry values of 48 mmHg and 52 mmHg recorded in the present Siberian tiger, comparison with published feline intraocular-pressure reference data obtained with the same class of instrument is informative. In clinically normal domestic cats (*Felis catus*), Rusanen et al. [21] established a mean IOP of 20.74 mmHg using the TonoVet^®^ rebound tonometer in a cohort of 100 cats, with a concurrent applanation-tonometer mean of 18.4 mmHg; the authors explicitly reported that rebound-tonometry values exceeded applanation values by 2–3 mmHg, a systematic offset within clinically acceptable limits. Kovalcuka et al. [22] subsequently reported a TonoVet^®^ mean IOP of 18.88 ± 3.98 mmHg in 20 clinically healthy cats. The TonoVet^®^ has also been validated against direct manometry in a cannulation study performed in normal and glaucomatous cats across the clinically relevant range of 5–70 mmHg, with readings generally 2–5 mmHg higher than true manometric pressure [23], a finding consistent with the newer TONOVET Plus^®^ data reported for normal and glaucomatous cats [24].

Even after accounting for the typical 2–5 mmHg positive offset of rebound tonometry in felids, the IOP values recorded in the present case exceeded all published normal feline and adult-tiger reference ranges by more than twofold and were therefore consistent with severe glaucoma. Terminally elevated glaucomatous IOPs in the range of 70–90 mmHg have been reported in the two published tiger cases [17,18], and buphthalmic, blind end-stage globes with secondary glaucoma have been reported in lions [19,20]. The physiological IOP range of the Siberian tiger measured by rebound tonometry remains to be established.

In veterinary ophthalmology, glaucoma is classified as primary or secondary based on the underlying cause of impaired aqueous humor outflow, and as open-angle or closed-angle based on the morphology of the iridocorneal angle [2]. In the present case, the absence of prior ocular disease, such as chronic uveitis, intumescent cataract, or intraocular neoplasia, supported a primary etiology. Biomicroscopy performed with a portable slit lamp and B-mode ultrasonography suggested that the entry to the ciliary cleft remained open. In the absence of direct gonioscopic evaluation, this finding, together with the elevated intraocular pressure and fundic changes, supported the diagnosis of open-angle glaucoma.

The clinical diagnosis was well supported by the markedly elevated intraocular pressure, irreversible bilateral blindness, bilateral buphthalmos, and extensive retinopathy, all consistent with advanced glaucomatous disease inferred from functional and indirect structural evidence; however, in vivo determination of the exact morphological subtype of the glaucoma was subject to methodological limitations. In the absence of ultrasound biomicroscopy (UBM), analysis of the microstructure of the iridocorneal angle (e.g., subtle changes in the trabecular meshwork or the pectinate ligament) was not feasible. High-frequency ultrasound and UBM can provide detailed visualization of the iridocorneal angle and have become established non-invasive modalities in domestic species [16]; their application in non-domestic felids, however, is largely limited by the need for specialized equipment. The pathogenesis of primary open-angle glaucoma typically involves a gradual increase in aqueous humor outflow resistance within the trabecular meshwork, often attributed to biochemical or ultrastructural changes in the extracellular matrix. In tigers maintained under human care, genetic predisposition cannot be excluded, as the process is often bilateral and slowly progressive prior to reaching the advanced stage observed in this individual.

The mild lens opacities observed during ophthalmoscopy were not judged to be of significant visual consequence. The bilateral extensive retinopathy with a map-like pattern, together with the focal degenerative lesion in the left eye, are most consistent with advanced glaucomatous retinal damage. Chronically elevated intraocular pressure produces RGC loss and secondary retinal vascular and pigmentary changes, as reviewed in the feline glaucoma literature [2]. Oral examination revealed no pathological findings that would have supported an infectious or inflammatory systemic differential; this finding, together with the absence of ocular signs suggestive of uveitis, such as aqueous flare, keratic precipitates, posterior synechiae, or ocular hypotony, is consistent with the interpretation of the disease as primary, rather than secondary, glaucoma.

Glaucoma is increasingly recognized as a neurodegenerative disease characterized by primary damage to the RGC axons at the level of the optic nerve head, followed by retrograde axonal degeneration and subsequent apoptotic loss of the RGCs, together resulting in irreversible optic neuropathy and visual loss [25,26]. Although elevated intraocular pressure remains the most significant modifiable risk factor, the cellular pathways implicated in disease progression are multifactorial and include impaired axonal transport, cellular hypoxia, disturbances of local blood supply, and excitotoxicity associated with glutamate accumulation [26]. In principle, early structural detection of RGC loss or dysfunction is possible by imaging of the optic disk and retinal nerve fiber layer, as discussed in the general glaucoma literature [25], and by electroretinography (ERG), which provides an objective record of retinal function and is an established clinical modality in veterinary ophthalmology [27]. Spectral-domain optical coherence tomography (OCT) would have permitted non-invasive in vivo quantification of the peripapillary retinal nerve fiber layer and optic nerve head morphology [25,26]. However, none of these modalities were available under the cage-side field conditions of the present case, because of the distance between the examination site and the nearest equipped veterinary facility.

Recognizing glaucomatous optic nerve head cupping by ophthalmoscopy was particularly difficult in this case owing to the distinctive anatomy of the felid optic nerve head. In domestic cats—and, by extension, in other felids—myelination of the RGC axons does not begin until posterior to the lamina cribrosa, and the disk normally sits at, or slightly below, the level of the peripapillary retina, so that a physiological excavation comparable to that of the human or canine optic disk is absent. The recognition of early glaucomatous excavation is therefore considerably more difficult than in dogs, in which the prelaminar myelin gives the disk a raised appearance [2]. The subtle ophthalmoscopic signs that may indicate advanced feline glaucomatous optic neuropathy—notably a peripapillary pigmented or hyper-reflective halo and increased prominence of the laminar pores, the so-called “laminar dot sign”—were not clearly discernible on the fundus photographs recorded in the present case, most probably because of the extensive retinopathy and mild media opacities already described, as well as the species-specific optic nerve head anatomy of felids outlined above [2]. Nevertheless, the chronically and markedly elevated intraocular pressure documented in both eyes, measuring 48–52 mmHg at examination and consistently above the feline reference range in previous measurements, together with bilateral buphthalmos, irreversible blindness, fixed dilated pupils, and absent menace responses, strongly supports the diagnosis of advanced glaucomatous optic neuropathy [2,21,22,23,24].

The therapeutic evidence base for glaucoma in large felids is limited, but scientific case reports indicate that both medical and surgical management have been attempted. Unilateral secondary glaucoma in a circus-rescued male tiger, attributed to training-related ocular trauma, has been documented under sedation [17], and unilateral congenital glaucoma in an 8-month-old female tiger was successfully managed by transconjunctival enucleation [18]; diffuse iris melanoma with secondary glaucoma in an adult African lion was likewise treated by enucleation [20]. The broader therapeutic framework for feline glaucoma, comprehensively reviewed for the domestic cat [2], is largely applicable by extension to non-domestic felids. Medical management relies primarily on topical carbonic anhydrase inhibitors (e.g., dorzolamide) and β-adrenergic antagonists (e.g., timolol) to reduce aqueous-humor production; prostaglandin F_2_α analogs are typically ineffective or ocularly irritating in cats [2]. In non-domestic felids under human care, however, the frequent topical administration required is often impracticable without intensive operant-conditioning training, and repeated close handling carries a substantial occupational-safety risk [7]. Among vision-preserving surgical procedures, laser cyclophotocoagulation and anterior-chamber shunts are less extensively reported in cats than in dogs and appear less effective in felids than in canids, a difference that may in part reflect species-related variation in ciliary-body pigmentation and architecture [2]. In irreversibly blind or painful eyes, enucleation and evisceration with an intrascleral silicone prosthesis are the two established advanced-stage options. In cats, enucleation is generally preferred because of the possibility of occult intraocular neoplasia and the comparatively higher failure rate of intrascleral prostheses [2], and both enucleation and evisceration have been successfully performed in tigers and other large felids [17,18,20].

The complete absence of direct and consensual pupillary light reflexes observed in the examined individual is consistent with severe damage to the visual pathways, including the retina and optic nerve, resulting from advanced glaucoma. Although functional blindness can be associated with persistent mydriasis, the fixed pupillary dilation recorded during the anesthesia was probably also influenced by the pharmacological action of ketamine, a non-competitive N-methyl-D-aspartate (NMDA)-receptor antagonist that activates central sympathetic outflow and the limbic system, thereby modulating pupillary tone in felids. Ketamine may also contribute to mydriasis through inhibition of muscarinic acetylcholine receptors [7,12]. In felids, the effect of the α_2_-adrenergic agonist medetomidine to pupillary size is variable, and mydriasis has been reported in cats after α_2_-agonist administration [8]. The effects of the medetomidine–ketamine–butorphanol combination on intraocular pressure and physiological parameters were carefully considered throughout the immobilization protocol [10,11].

Beyond the glaucomatous ocular pathology, the clinical picture in this case included neurological features that warrant separate consideration. The severe bilateral convergent strabismus (esotropia) recorded during remote observation is of considerable diagnostic significance. This sign is not a direct consequence of glaucomatous ocular hypertension: in veterinary neurology it is classically attributed to dysfunction of the abducens nerve (cranial nerve VI), whose lateral-rectus motor output normally antagonizes the medial rectus. Bilateral abducens nerve dysfunction may therefore produce bilateral esotropia and is a recognized non-localizing sign of raised intracranial pressure because of the long subarachnoid course of the nerve [9]. In combination with disorientation and the seizure activity documented in the present case, this finding raises the differential diagnoses of a space-occupying intracranial process, meningoencephalitis, or generalized intracranial hypertension; however, without advanced cross-sectional neuroimaging, none of these conditions can be confirmed or excluded.

The coexistence of bilateral convergent strabismus and the recorded epileptic episodes suggest possible involvement of the central nervous system, whose exact etiology cannot be clarified without advanced cross-sectional imaging. Magnetic resonance imaging (MRI) is generally considered a key diagnostic modality for the differentiation of inflammatory, neoplastic, vascular, and degenerative intracranial disease in both domestic and non-domestic felids. Hecht et al. [28] reported the largest MRI case series to date in non-domestic *Felidae* under human care, comprising 50 animals, including 18 tigers, 11 lions, and 4 leopards, and documenting Chiari-like malformation, meningoencephalitis, pituitary lesions, leukoencephalopathy, and vascular events among the most frequent intracranial diagnoses. That same series also illustrates the considerable logistical and anesthetic demands of performing MRI in large non-domestic felids and the limited availability of such examinations outside specialized academic institutions [28]. In the absence of CT or MRI in the present case, a causal relationship between the glaucomatous blindness and the neurological signs—for example, a lesion involving the optic chiasm, leukoencephalopathy, or another space-occupying process—remains speculative, and other congenital or acquired intracranial anomalies cannot be formally excluded.

**Figure 5:**
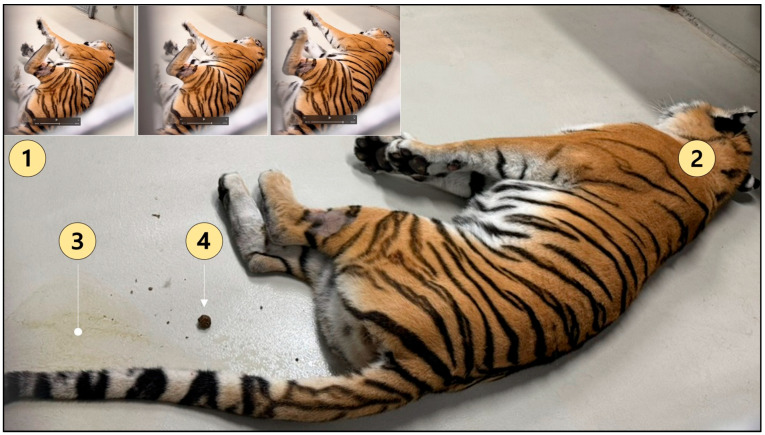
Generalized tonic–clonic seizure during recovery from anesthesia (1), with lateral recumbency (2) and urinary (3) and fecal (4) incontinence, immediately after completion of the examination.

**Figure 6:**
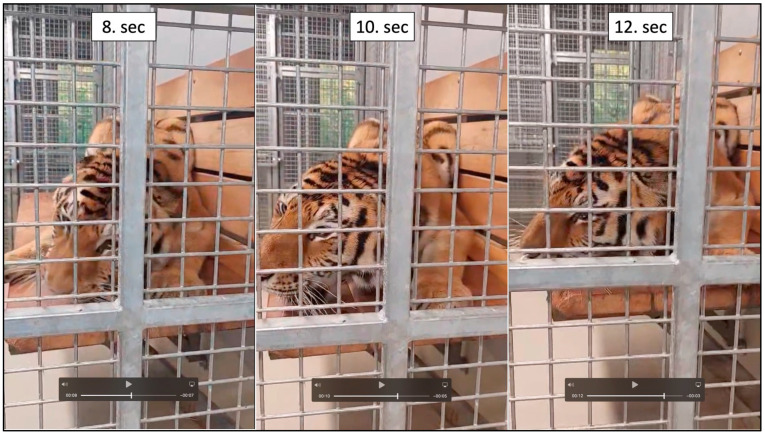
Brief stereotyped episode on the day following the examination, clinically consistent with a focal seizure rather than true behavioral rage (no clawing or paw-striking; preserved postural tone; duration approximately 15 s).

Two distinct types of seizure-like episodes were observed: brief, stereotyped focal episodes reported by the keepers since the animal’s arrival at the zoo four months before anesthesia, and generalized tonic–clonic seizures observed only during anesthetic recovery (Figure 5). A representative focal episode also documented on the day after the examination (Figure 6). These two event types are not mutually exclusive in their interpretation: the recovery-phase generalized seizures may have represented secondary generalization of an underlying chronic focal epileptogenic process, facilitated by the combined effects of residual ketamine and premature α_2_-adrenoceptor antagonism. The interval between the final ketamine dose (T = 32 min) and atipamezole administration (T = 59 min) was 27 min; in the established veterinary literature on the medetomidine–ketamine–atipamezole protocol in non-domestic mammals, premature antagonism of the α_2_-adrenoceptor agonist before residual ketamine has been substantially metabolized is recognized as a cause of rough recoveries with muscle rigidity, tremor, and, less commonly, excitatory phenomena [11,29]. Experimental evidence in the domestic cat further indicates that ketamine activates the limbic system and can induce clinical seizure activity closely resembling an animal’s habitual spontaneous seizure pattern when a pre-existing epileptogenic substrate is present, a phenomenon potentially relevant to this case [13].

Qualitatively, ketamine-related myoclonic or tremor-like movements described during recovery from dissociative anesthesia are typically brief, self-limiting, and clinically distinguishable from both the generalized tonic–clonic seizures and the stereotyped focal episodes observed here [7,11,13,16]. The decision to withhold emergency anti-seizure treatment during the recovery phase was a deliberate clinical choice based on the short, self-limiting duration of the episodes, the continued presence of residual sedative effect, the practical constraints of operating within an outdoor enclosure shared with other large felids, and the substantial occupational-safety risk of invasive drug administration in this setting; neither the recovery-phase episodes nor the focal seizure observed on the following day met the accepted clinical criteria for status epilepticus or cluster seizures [30]. A chronic, active inflammatory central nervous system disease—for which published imaging series in non-domestic felids report characteristic findings typically accompanied by progressive weight loss, hyporexia, and deteriorating body condition [28]—was considered unlikely in this animal, which maintained excellent body condition, normal appetite, and stable activity throughout the four-month observation period before anesthesia. For future immobilization of large felids with suspected or documented neurological comorbidity, a longer interval (ideally ≥45–60 min) between the final ketamine dose and α_2_-adrenoceptor antagonist administration, lower incremental ketamine doses where clinically acceptable, or alternative protocols such as medetomidine–midazolam–butorphanol or butorphanol–azaperone–medetomidine with low-dose ketamine should be considered [2,6,10,11].

In the present case, the combination of bilateral, irreversible blindness, the absence of clinically evident ocular pain or self-directed behavior, and the logistical and safety constraints of frequent topical therapy or repeated general anesthesia in an adult tiger held in a small private zoo led the attending team to shift the therapeutic goal from vision preservation to long-term pain surveillance and welfare maintenance. The veterinary recommendations issued after the examination were therefore as follows: (i) adaptation of the housing environment to a simplified, enclosed indoor enclosure with a consistent layout, familiar olfactory cues, and protected feeding and watering stations, in order to minimize injury risk associated with blindness; (ii) referral to a facility equipped with advanced cross-sectional neuroimaging, including CT and MRI, electroencephalography, and specialist ophthalmic surgery, to permit both etiological characterization of the focal-seizure phenomena and definitive surgical management of the bilateral advanced-stage glaucoma; (iii) consideration of long-term systemic analgesia pending definitive surgery or if referral was not feasible, with explicit account taken of the occupational-safety implications of repeated handling; and (iv) ongoing monitoring of neurological signs and welfare indicators by the holding-zoo veterinary team, with a clearly defined threshold for escalation to humane euthanasia should the welfare balance deteriorate [2,7].

## 4. Conclusions

This case report highlights that glaucoma, although rarely diagnosed in big cats, can be a significant underlying cause of atypical behavior and profound vision loss in non-domestic felids under human care. In the four-year-old Siberian tiger (*Panthera tigris altaica*) examined here, markedly and bilaterally elevated intraocular pressure (48–52 mmHg), bilateral buphthalmos, absent pupillary light and menace responses, extensive retinopathy, and focal retinal degeneration together supported the diagnosis of advanced-stage bilateral primary glaucoma with irreversible vision loss.

The concurrent presence of severe bilateral convergent strabismus and both generalized tonic–clonic and focal seizure episodes indicated that the clinical picture could not be explained by the ocular pathology alone, and was most consistent with an additional, pre-existing central nervous system process, such as chronically elevated intracranial pressure, a space-occupying lesion, or a focal epileptogenic lesion, acting independently of, or in synergy with, the advanced-stage glaucoma. Definitive etiological characterization of the neurological component would have required advanced cross-sectional neuroimaging and electroencephalography, which were not available at the holding facility. The diagnosis of bilateral, irreversible glaucomatous blindness was nonetheless sufficient to support the clinical conclusions and subsequent case management.

Because vision could not be restored, the therapeutic goal shifted from vision preservation to long-term pain surveillance and welfare maintenance. The case underscores the importance of considering painful ocular disease in the differential diagnosis of behavioral and neurological abnormalities in large felids under human care, and highlights the need for accessible on-site or readily available referral-based ophthalmic and cross-sectional imaging capability in zoological institutions, so that severe ocular conditions in large exotic felids can be recognized and managed in a timely, welfare-oriented manner.

## Data Availability

The original contributions presented in this study are included in the article/Supplementary Materials. Further inquiries can be directed to the corresponding author.

## Supplementary Materials

The following supporting information can be downloaded at: https://www.mdpi.com/article/10.3390/ani16111647/s1, Video S1: Behavior before examination; Video S2: Anesthetic dart; Video S3: Generalized tonic–clonic seizure; Video S4: A stereotyped focal seizure-like episode after examination.

## Abbreviations

The following abbreviations are used in this manuscript:

ERG	Electroretinography
IM	Intramuscular
IOP	Intraocular pressure
mmHg	Millimeters of mercury (unit of pressure)
MRI	Magnetic resonance imaging
NMDA	N-methyl-D-aspartate
OCT	Optical Coherence Tomography
PLR	Pupillary light reflex
POAG	Primary open-angle glaucoma
RGC	Retinal ganglion cell
UBM	Ultrasound biomicroscopy
